# Deep Graph Mapper: Seeing Graphs Through the Neural Lens

**DOI:** 10.3389/fdata.2021.680535

**Published:** 2021-06-16

**Authors:** Cristian Bodnar, Cătălina Cangea, Pietro Liò

**Affiliations:** Department of Computer Science and Technology, University of Cambridge, Cambridge, United Kingdom

**Keywords:** mapper, graph neural networks, pooling, graph summarization, graph classification

## Abstract

Graph summarization has received much attention lately, with various works tackling the challenge of defining pooling operators on data regions with arbitrary structures. These contrast the grid-like ones encountered in image inputs, where techniques such as max-pooling have been enough to show empirical success. In this work, we merge the Mapper algorithm with the expressive power of graph neural networks to produce topologically grounded graph summaries. We demonstrate the suitability of Mapper as a topological framework for graph pooling by proving that Mapper is a generalization of pooling methods based on soft cluster assignments. Building upon this, we show how easy it is to design novel pooling algorithms that obtain competitive results with other state-of-the-art methods. Additionally, we use our method to produce GNN-aided visualisations of attributed complex networks.

## 1 Introduction

The abundance of relational information in the real world and the success of deep learning techniques have brought renowned interest in learning from graph-structured data. Efforts in this direction have been primarily focused on replicating the hierarchy of convolutional filters and pooling operators, which have achieved previous success in computer vision Sperduti. (1994); Goller and Kuchler. (1996); Gori et al. (2005); Scarselli et al. (2009); Bruna et al. (2014); Li et al. (2015), within relational data domains. In contrast to image processing applications, where the signal is defined on a grid-like structure, designing graph coarsening (pooling) operators is a much more difficult problem, due to the arbitrary structure typically present in graphs.

In this work, we introduce Structural Deep Graph Mapper (SDGM)^1^—an adaptation of Mapper (Singh et al., 2007), an algorithm from the field of Topological Data Analysis (TDA) (Chazal and Michel, 2017), to graph domains. First, we prove that SDGM is a generalization of pooling methods based on soft cluster assignments, which include state-of-the-art algorithms like minCUT (Bianchi et al., 2019) and DiffPool (Ying et al., 2018). Building upon this topological perspective of graph pooling, we propose two pooling algorithms leveraging fully differentiable and fixed PageRank-based “lens” functions, respectively. We demonstrate that these operators achieve results competitive with other state-of-the-art pooling methods on graph classification benchmarks. Furthermore, we show how our method offers a means to flexibly visualize graphs and the complex data living on them through a GNN “lens” function.

## 2 Related Work

In this section, we investigate the existing work in the two broad areas that our method is part of—graph pooling (also deemed hierarchical representation learning) and network visualisations.

### 2.1 Graph Pooling

Algorithms have already been considerably explored within GNN frameworks for graph classification. Luzhnica et al. (2019) propose a topological approach to pooling, which coarsens the graph by aggregating its maximal cliques into new clusters. However, cliques are local topological features, whereas our methods leverage a global perspective of the graph during pooling. Two paradigms distinguish themselves among learnable pooling layers: Top-*k* pooling based on a learnable ranking (Gao and Ji, 2019), and learning the cluster assignment (Ying et al., 2018) with additional entropy and link prediction losses for more stable training (DiffPool). Following these two trends, several variants and incremental improvements have been proposed. The Top-*k* approach is explored in conjunction with jumping-knowledge networks (Cangea et al., 2018), attention (Huang et al., 2019; Lee et al., 2019) and self-attention for cluster assignment (Ranjan et al., 2019). Similarly to DiffPool, the method suggested by Bianchi et al. (2019) uses several loss terms to enforce clusters with strongly connected nodes, similar sizes and orthogonal assignments. A different approach is also proposed by Ma et al. (2019), who leverage spectral clustering.

### 2.2 Graph Visualization

Graph visualization is a vast topic in network science. We therefore refer the reader to existing surveys, for a complete view of the field (Nobre et al., 2019; von Landesberger et al., 2011; Beck et al., 2017), and focus here only on methods that, similarly to ours, produce node-link-based visual summaries through the aggregation of static graphs. Previous methods rely on grouping nodes into a set of predefined motifs (Dunne and Shneiderman, 2013), modules (Dwyer et al., 2013) or clusters with basic topological properties (Batagelj et al., 2010). Recent approaches have considered attribute-driven aggregation schemes for multivariate networks. For instance, PivotGraph (Wattenberg, 2006) groups the nodes based on categorical attributes, while van den Elzen and van Wijk. (2014) propose a more sophisticated method using a combination of manually specified groupings and attribute queries. However, these mechanisms are severely constrained by the simple types of node groupings allowed and the limited integration between graph topology and attributes. Closest to our work, Mapper-based summaries for graphs have recently been considered by Hajij et al. (2018). Despite the advantages provided by Mapper, their approach relies on hand-crafted graph-theoretic “lenses,” such as the average geodesic distance, graph density functions or eigenvectors of the graph Laplacian. Not only are these functions unable to fully adapt to the graph of interest, but they are also computationally inefficient and do not take into account the attributes of the graph.

## 3 Background and Formal Problem Statement

### 3.1 Formal Problem Statement

Consider a dataset whose samples are formed by a graph $G_{i}=\left(V_{i},E_{i}\right)$, A *d*-dimensional signal defined over the nodes of the graph $h_{i}:V\to {\mathbb{R}}^{d}$ and a label $y_{i}$ associated with the graph, where i∈I, a finite indexing set for the dataset samples. We are interested in the setting where graph neural networks are used to classify such graphs using a sequence of (graph) convolutions and pooling operators. While convolutional operators act like filters of the graph signal, pooling operators coarsen the graph and reduce its spatial resolution. Unlike image processing tasks, where the inputs exhibit a regular grid structure, graph domains pose challenges for pooling. In this work, we design topologically inspired pooling operators based on Mapper. As an additional contribution, we also investigate graph pooling as a tool for the visualization of attributed graphs.

We briefly review the Mapper (Singh et al., 2007) algorithm, with a focus on graph domains (Hajij et al., 2018). We first introduce the required mathematical background.

Definition 3.1: Let X,Z be two topological spaces, f:X→Z, a continuous function, and $U={\left(U_{i}\right)}_{i\in I}$ a cover of *Z*. Then, the pull back cover $f^{-1}\left(U\right)$ of *X* induced by (f,U) is the collection of open sets $f^{-1}\left(U_{i}\right),i\in I$, for some indexing set *I*. For each $f^{-1}\left(U_{i}\right)$, let ${\left\{C_{i,j}\right\}}_{j\in J_{i}}$ be a partition of $f^{-1}\left(U_{i}\right)$ indexed by $J_{i}$. We refer to the elements of these partitions as clusters. The resulting collection of clusters forms another cover of *X* called the refined pull back cover $ℛ\left(f^{-1}\left(U\right)\right)={\left\{C_{i,j}\right\}}_{i\in I,j\in J_{i}}$.

Definition 3.2: Let *X* be a topological space with an open cover $U={\left(U_{i}\right)}_{i\in I}$. The 1-skeleton of the nerve N(U) of U, which we denote by ${\text{sk}}_{1}\left(N\left(U\right)\right)$, is the graph with vertices given by ${\left(v_{i}\right)}_{i\in I}$, where two vertices $v_{i},v_{j}$ are connected if and only if $U_{i}\cap U_{j}\neq \emptyset$.

### 3.2 Mapper

Given a topological space *X*, a carefully chosen lens function f:X→Z and a cover U of *Z*, Mapper produces a graph representation of the topological space by computing the 1-skeleton of the nerve of the refined pull back cover ${\text{sk}}_{1}\left(N\left(ℛ\left(f^{-1}\left(U\right)\right)\right)\right)$, which we denote by ℳ(f,U). We note that, more generally, the skeleton operator might be omitted, in which case the output of the algorithm becomes a simplicial complex. However, for the purpose of this work, we are only interested in graph outputs. Typically, the input to the Mapper algorithm is a point cloud and the connected components are inferred using a statistical clustering algorithm, with the help of a metric defined in the space where the points live.

**Mapper for Graphs**. More recently, Hajij et al. (2018) considered the case when the input topological space X=G(V,E) is a also a graph with vertices *V* and edge set *E*. In a typical point cloud setting, the relationships between points are statistically inferred; in a graph setting, the underlying relationships are given by the edges of the graph. The adaptation of Mapper for graphs proposed by Hajij et al. (2018) uses a lens function f:V→ℝ based on graph-theoretic functions and a cover U formed of open intervals of the real line. Additionally, the connected components ${\left\{C_{i,j}\right\}}_{j\in J_{i}}$ are given by the vertices of the connected components of the subgraph induced by $f^{-1}\left(U_{i}\right)$.

However, the graph version of Mapper described above has two main limitations. Firstly, the graph-theoretic functions considered for *f* are rather limited, not taking into account the signals which are typically defined on the graph in signal processing tasks, such as graph classification. Secondly, by using a pull back cover only over the graph vertices, as opposed to a cover of the entire graph, the method relies exclusively on the lens function to capture the structure of the graph and the edge-connections between the clusters. This may end up discarding valuable structural information, as we later show in Section 7.7.

## 4 Structural Deep Graph Mapper

Structural Graph Mapper. One of the disadvantages of the graph version of Mapper (described in the background section) is that its output does not explicitly capture the connections between the resulting collections of clusters. This is primarily because the lens function *f* is defined only over the set of vertices *V* and, consequently, the resulting pull-back cover only covers *V*. In contrast, one should aim to obtain a cover for the graph *G*, which automatically includes the edges. While this could be resolved by considering a lens function over the geometric realization of the graph, handling only a finite set of vertices is computationally convenient.

To balance these trade-offs, we add an extra step to the Mapper algorithm. Concretely, we extend the refined pull back cover into a cover over both nodes and edges. Given the set of refined clusters ${\left\{C_{i,j}\right\}}_{i\in I,j\in J_{i}}$, we compute a new set of clusters ${\left\{C\prime_{i,j}\right\}}_{i\in I,j\in J_{i}}$ where each cluster $C\prime_{i,j}$ contains the elements of $C_{i,j}$ as well as all the edges incident to the vertices in $C_{i,j}$. We use ${ℛ}_{E}$ (the edge-refined pull back cover) to refer to this open cover of the graph *G* computed from $f^{-1}\left(U\right)$. Then, our algorithm can be written as ${\text{sk}}_{1}\left(N\left({ℛ}_{E}\left(f^{-1}\left(U\right)\right)\right)\right)$ and we denote it by GM(f,U).

Remark 1: We note that Structural Mapper, unlike the original Mapper method, encodes two types of relationships via the edges of the output graph. The semantic connections highlight a similarity between clusters, according to the lens function (that is, two clusters have common nodes—as before), while structural connections show how two clusters are connected (namely, two clusters have at least one edge in common). This latter type of connection is the result of considering the extended cover over the edges. The two types of connections are not mutually exclusive because two clusters might have both nodes and edges in common.

 We now broadly outline our proposed method, using the three main degrees of freedom of the Mapper algorithm to guide our discussion: the lens function, the cover, and the clustering algorithm.

### 4.1 Lens

The lens is a function $f:V\to {\mathbb{R}}^{d}$ over the vertices, which acts as a filter that emphasizes certain features of the graph. Typically, d is a small integer—in our case, d∈{1,2}. The choice of *f* depends on the graph properties that should be highlighted by the visualization. In this work, we leverage the recent progress in the field of graph representation learning and propose a parameterized lens function based on graph neural networks (GNNs). We thus consider a function $f_{\theta }\left(v\right)=g_{\theta }{\left(V,E,X\right)}_{v}$, where *g* is a GNN with parameters *θ* taking as input a graph G=(V,E) with *n* nodes and node features $X\in {\mathbb{R}}^{n\times k}$. For visualization purposes, we often consider a function composition $f_{\theta }\left(v\right)={\left(r\circ g_{\theta }\right)}_{v}$, where $r:{\mathbb{R}}^{n\times d^{\prime }}\to {\mathbb{R}}^{n\times d}$ is a dimensionality reduction algorithm like *t*-SNE (van der Maaten and Hinton, 2008).

Unlike the traditional graph theoretic lens functions proposed by Hajij et al. (2018), GNNs can naturally learn to integrate the features associated with the graph and its topology, while also scaling computationally to large, complex graphs. Additionally, visualisations can be flexibly tuned for the task of interest, by adjusting the lens $g_{\theta }$ through the loss function of the model.

### 4.2 Cover

The cover U determines the resolution of the output graph. For most purposes, we leverage the usual cover choice for Mapper, ${\mathbb{R}}^{d}$. When d=1, we use a set of equally sized overlapping intervals over the real line. When d=2, this is generalized to a grid of overlapping cells in the real plane. Using more cells will produce more detailed visualisations, while higher overlaps between the cells will increase the connectivity of the output graph. When chosen suitably, these hyperparameters are a powerful mechanism for obtaining multi-scale visualisations.

Another choice that we employ for designing differentiable pooling algorithms is a set of RBF kernels, where the second arguments of kernel functions are distributed over the real line. We introduce this in detail in Section 5.2.

### 4.3 Clustering

Clustering statistically approximates the (topological) connected components of the cover sets $U_{i}$. Mapper does not require a particular type of clustering algorithm; however, when the input topological space *X* is a graph, a natural choice, also adopted by Hajij et al. (2018), is to take the connected components of the subgraphs induced by the vertices $f^{-1}\left(U_{i}\right),i\in I$. Therefore, in principle, there is no need to resort to statistical clustering techniques.

However, relying on the topological connected components introduces certain challenges when the aim is to obtained a coarsened graph. Many real-world graphs comprise thousands of connected components, which is a lower bound to the number of connected components of the graph produced by GM. In the most extreme case, a graph containing only isolated nodes (namely, a point cloud) would never be coarsened by this procedure. Therefore, it is preferable to employ statistical techniques where the number of clusters can be specified. In our pooling experiments, we draw motivation from the relationship with other pooling algorithms and opt to assign all the nodes to the same cluster (which corresponds to no clustering).

**FIGURE 1:**
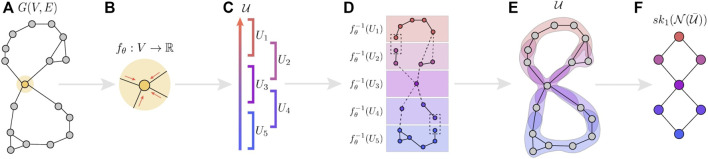
A cartoon illustration of Structural Deep Graph Mapper (SDGM) where, for simplicity, a graph neural network (GNN) approximates a “height” function over the nodes in the plane of the diagram. The input graph **(A)** is passed through the GNN, which maps the vertices of the graph to a real number (the height) **(B–C)**. Given a cover U of the image of the GNN **(C)**, the edge-refined pull back cover $\bar{U}$ is computed **(D–E)**. The dotted edges in **(D)** illustrate connections between the node clusters (strucutal connections), while the dotted boxes show nodes that appear in multiple clusters (semantic connections). The 1-skeleton of the nerve of the edge-refined pull back cover provides the pooled graph **(F)**.

We broadly refer to this instance of Structural Graph Mapper, with the choices described above, as Structural Deep Graph Mapper (SDGM). We summarize it step-by-step in the cartoon example in Figure 1 and encourage the reader to refer to it.

## 5 Structural Graph Mapper for Pooling

We begin this section by introducing several theoretical results, which provide a connection between our version of Mapper and other graph pooling algorithms. We then use these results to show how novel pooling algorithms can be designed.

### 5.1 Relationship to Graph Pooling Methods

An early suggestion that Mapper could be suitable for graph pooling is given by the fact that it constitutes a generalization of binary spectral clustering, as observed by Hajij et al. (2018). This link is a strong indicator that Mapper can compute “useful” clusters for pooling. We formally restate this observation below and provide a short proof.

Proposition 5.1: Let *L* be the Laplacian of a graph G(V,E) and $l_{2}$ the eigenvector corresponding to the second lowest eigenvalue of *L*, also known as the Fiedler vector (Fiedler, 1973). Then, for a function $f:V\to \mathbb{R},f\left(v\right)=l_{2}\left(v\right)$, outputting the entry in the eigenvector $l_{2}$ corresponding to node *v* and a cover U={(−∞,ε),(−ε,+∞)}, Mapper produces a spectral bi-partition of the graph for a sufficiently small positive *ϵ*.

 *Proof*: It is well known that the Fiedler vector can be used to obtain a “good” bi-partition of the graph based on the signature of the entries of the vector (i.e., $l_{2}\left(v\right)>0$ and $l_{2}\left(v\right)<0$) (please refer to Demmel. (1995) for a proof). Therefore, by setting *ϵ* to a sufficiently small positive number $\varepsilon <\min_{v}\left|l_{2}\left(v\right)\right|$, the obtained pull back cover is a spectral bi-partition of the graph.

 The result above indicates that Mapper is a generalization of spectral clustering. As the latter is strongly related to min-cuts (Leskovec, 2016), the proposition also links them to Mapper. We now provide a much stronger result in that direction, showing that Structural Mapper is a generalization of all pooling methods based on soft-cluster assignments. Soft cluster assignment pooling methods use a soft cluster assignment matrix $S\in {\mathbb{R}}^{N\times K}$, where $S_{ij}$ encodes the probability that node *i* belongs to cluster *j*, *N* is the number of nodes in the graph and *K* is the number of clusters. The adjacency matrix of the pooled graph is computed via $A^{\prime }=S^{T}\left(A+I\right)S$. Below, we prove a helpful result concerning this class of methods.

Lemma 5.1: The adjacency matrix $A^{\prime }=S^{T}\left(A+I\right)S$ defines a pooled graph, where the nodes corresponding to clusters encoded by S are connected if and only if there is a common edge (including self-loops) between them.

 *Proof:* Let L=AS. Then, $A_{ij}^{\prime }=\sum_{k}^{N}S_{ik}^{T}L_{kj}=0$ if and only if $S_{ik}^{T}=0$ (node *k* does not belong to cluster *i*) or $L_{kj}=0$ (node *k* is not connected to any node belonging to cluster *j*), for all *k*. Therefore, $A_{ij}^{\prime }\neq 0$ if and only if there exists a node *k* such that *k* belongs to cluster *i* and *k* is connected to a node from cluster *j*. Due to the added self-loops, $A_{ij}^{\prime }\neq 0$ also holds if there is a node *k* belonging to both clusters.

Proposition 5.2: GM(f,U) generalizes approaches based on soft-cluster assignments.

 *Proof:* Let $s:V\to {△}_{K-1}$ be a soft cluster assignment function that maps the vertices to the (K−1)-dimensional unit simplex. We denote by $s_{k}\left(v\right)$ the probability that vertex *v* belongs to cluster k≤K and $\sum_{k}^{K}s_{k}\left(v\right)=1$. This function can be completely specified by a cluster assignment matrix $S\in {\mathbb{R}}^{N\times K}$ with $S_{ik}=s_{k}\left(i\right)$. This is the soft cluster assignment matrix computed by algorithms like minCut and DiffPool. Let $U={\left\{U_{i}\right\}}_{i\leq K}$ with $U_{i}=\left\{x\in {△}_{K-1}x=\sum_{j}\lambda_{j}u_{j},\sum_{j}\lambda_{j}=1\;\text{and }\lambda_{i}\rangle 0\right\}$ be an open cover of ${△}_{K-1}$. Then consider an instance of GM where everything is assigned to a single cluster (i.e. same as no clustering). Clearly, there is a one-to-one correspondence between the vertices of GM(s,U) and the soft clusters. By Remark 1, the nodes corresponding to the clusters are connected only if the clusters share at least one node or at least one edge. Then, by **Lemma 5.1** the adjacency between the nodes of GM(s,U) are the same as those described by $A^{\prime }=S^{T}\left(A+I\right)S$. Thus, the two pooled graphs are isomorphic.

 We hope that this result will enable theoreticians to study pooling operators through the topological and statistical properties of Mapper (Dey et al., 2017; Carriere et al., 2018; Carrière and Oudot, 2018). At the same time, we encourage practitioners to take advantage of it and design new pooling methods in terms of a well-chosen lens function *f* and cover U for its image. To illustrate this idea and showcase the benefits of this new perspective over graph pooling methods, we introduce two Mapper-based operators.

### 5.2 Differentiable Mapper Pooling

The main challenge for making pooling via Mapper differentiable is to differentiate through the pull back computation. To address this, we replace the cover of *n* overlapping intervals over the real line, described in the previous section, with a cover formed of overlapping RBF kernels $\phi \left(x,x_{i}\right)=\exp\left(-{\left|\left|x-x_{i}\right|\right|}^{2}/\delta \right)$, evaluated at *n* fixed locations $x_{i}$. The overlap between these kernels can be adjusted through the scale *δ* of the kernels. The soft cluster assignment matrix S is given by the normalized kernel values:

$$S_{ij}=\frac{\phi \left(\sigma {\left(f_{\theta }\left(X_{l}\right)\right)}_{i},x_{j}\right)}{{\sum^{\text{​}}}_{j=1}^{n}\phi \left(\sigma {\left(f_{\theta }\left(X_{l}\right)\right)}_{i},x_{j}\right)},$$ (1)

where the lens function $f_{\theta }$ is a GNN layer, *σ* is a sigmoid function ensuring the outputs are in [0,1], and $X_{\text{l}}$ are the node features at layer *l*. Intuitively, the more closely a node is mapped to a location $x_{i}$, the more it belongs to cluster *i*. By **Proposition 5.2**, we can compute the adjacency matrix of the pooled graph as $S^{T}\left(A+I\right)S$; the features are given by $S^{T}X$. This method can also be thought as a version of DiffPool (Ying et al., 2018), where the low-entropy constraint on the cluster assignment distribution is topologically satisfied, since a point cannot be equally close to many other points on a line. Therefore, each node will belong only to a few clusters if the scale *δ* is appropriately set.

**FIGURE 2:**
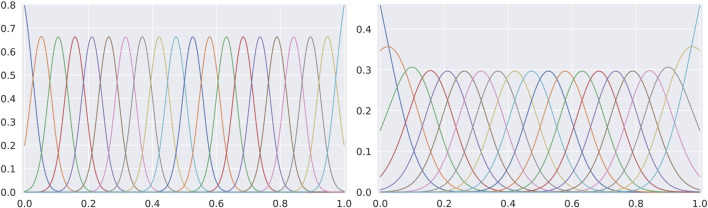
Two covers of RBF kernels with different scales: δ=0.002 and δ=0.01. The *x*-axis corresponds to the unit interval where the nodes of the graph are mapped. The *y*-axis represents the value of the normalized RBF kernels.

In Figure 2 we show two examples of RBF kernel covers for the output space. The scale of the kernel, *δ*, determines the amount of overlap between the cover elements. At bigger scales, there is a higher overlap between the clusters, as shown in the two plots. Because the line is one-dimensional, a point on the unit interval can only be part of a small number of clusters (that is, the kernels for which the value is greater than zero), assuming the scale *δ* is not too large. Therefore, DMP can be seen as a DiffPool variant where the low-entropy constraint on the cluster assignment is satisfied topologically, rather than by a loss function enforcing it.

### 5.3 Mapper-Based PageRank Pooling

To evaluate the effectiveness of the differentiable pooling operator, we also consider a fixed and scalable non-differentiable lens function f:V→ℝ that is given by the normalized PageRank (PR) (Page et al., 1999) of the nodes. The PageRank function assigns an importance value to each of the nodes based on their connectivity, according to the well-known recurrence relation:

$$f{\left(X\right)}_{i}\overset{\Delta }{=}PR_{i}=\sum_{j\in N\left(i\right)}\frac{PR_{j}}{\left|N\left(i\right)\right|},$$ (2)

where N(i) represents the set of neighbors of the *i*th node in the graph and the damping factor was set to the typical value of d=0.85. The resulting scores are values in [0,1] which reflect the probability of a random walk through the graph to end in a given node. Using the previously described overlapping intervals cover U, the elements of the pull back cover form a soft cluster assignment matrix S:

$$S_{ij}=\frac{I_{i\in f^{-1}\left(U_{j}\right)}}{\left|\left\{U_{k}|i\in f^{-1}\left(U_{k}\right)\right\}\right|}$$ (3)

where $U_{n}$ is the *n*th cover set in the cover U of [0,1]. It can be observed that the resulting clusters contain nodes with similar PageRank scores. Intuitively, this pooling method merges the (usually few) highly connected nodes in the graph, at the same time clustering the (typically many) dangling nodes that have a normalized PageRank score closer to zero. Therefore, this method favors the information attached to the most “important” nodes of the graph. The adjacency matrix of the pooled graph and the features are computed in the same manner as for DMP.

### 5.4 Model

For the graph classification task, each example G is represented by a tuple (X,A), where X is the node feature matrix and A is the adjacency matrix. Both our graph embedding and classification networks consist of a sequence of graph convolutional layers (Kipf and Welling, 2016); the *l*th layer operates on its input feature matrix as follows:

$$X_{\text{l}+1}=\sigma \left({\hat{D}}^{-\frac{1}{2}}\hat{A}{\hat{D}}^{-\frac{1}{2}}X_{\text{l}}W_{\text{l}}\right),$$ (4)

where $\hat{A}=A+I$ is the adjacency matrix with self-loops, $\hat{D}$ is the normalized node degree matrix, $W_{\text{l}}$ is the weight matrix of the *l*-th layer and *σ* is the activation function. After *E* layers, the embedding network simply outputs node features $X_{L_{E}}$, which are subsequently processed by a pooling layer to coarsen the graph. The classification network first takes as input node features of the Mapper-pooled graph,^2^ $X_{\text{MG}}$, and passes them through $L_{C}$ graph convolutional layers. Following this, the network computes a graph summary given by the feature-wise node average and applies a final linear layer which predicts the class:

$$y=\text{soft}\max\left(\frac{1}{\left|\text{MG}\right|}\sum_{i=1}^{\left|\text{MG}\right|}X_{L_{C}}W_{f}+b_{f}\right).$$ (5)

We note that either of these pooling operators could readily be adapted to the recently proposed message passing simplicial neural networks (MPSNs) (Bodnar et al., 2021) as a tool for coarsening simplicial complexes by dropping the 1-skeleton operator after computing the nerve. We leave this endeavor for future work.

### 5.5 Complexity

The topology of the output graph can be computed in O(V+E) time when using a cover over the unit interval, as described above. The output graph can be computed via (sparse) matrix multiplication given by $S^{T}\left(A+I\right)S$, to take advantage of GPU parallelism and compute the coefficients associated with the edges.

## 6 Pooling Experiments

### 6.1 Tasks

We illustrate the applicability of the Mapper-GNN synthesis within a pooling framework, by evaluating DMP and MPR in a variety of settings: social (IMDB-Binary, IMDB-Multi, Reddit-Binary, Reddit-Multi-5k), citation networks (Collab) and chemical data (D&D, Mutag, NCI1, Proteins) (Kersting et al., 2016).

### 6.2 Experimental Setup

We adopt a 10-fold cross-validation approach to evaluating the graph classification performance of DMP, MPR and other competitive state-of-the-art methods. The random seed was set to zero for all experiments (with respect to dataset splitting, shuffling and parameter initialisation), in order to ensure a fair comparison across architectures. All models were trained on a single Titan Xp GPU, using the Adam optimiser (Kingma and Ba, 2014) with early stopping on the validation set, for a maximum of 30 epochs. We report the classification accuracy using 95% confidence intervals calculated for a population size of 10 (the number of folds).

### 6.3 Baselines

We compare the performance of DMP and MPR to two other pooling methods that we identify mathematical connections with: minCUT (Bianchi et al., 2019) and DiffPool (Ying et al., 2018). Additionally, we include Graph U-Net (Gao and Ji, 2019) in our evaluation, as it has been shown to yield competitive results while performing pooling from the perspective of a learnable node ranking; we denote this approach by Top-*k* in the remainder of this section. The non-pooling baselines evaluated are the WL kernel (Shervashidze et al., 2011), a “flat” model (2 MP steps and global average pooling) and an average-readout linear classifier.

We optimize both DMP and MPR with respect to the cover cardinality *n*, the cover overlap (*δ* for DMP, overlap percentage *g* for MPR), learning rate and hidden size. The Top-*k* architecture is evaluated using the code provided in the official repository, where separate configurations are defined for each of the benchmarks. The minCUT architecture is represented by the sequence of operations described by Bianchi et al. (2019): MP(32)-pooling-MP(32)-pooling-MP(32)-GlobalAvgPool, followed by a linear softmax classifier. The MP(32) block represents a message-passing operation performed by a graph convolutional layer with 32 hidden units:

X(t+1)=ReLU(A˜X(t)Wm+X(t)Ws), (6)

where $\tilde{A}=D^{⁻1/2}AD^{⁻1/2}$ is the symmetrically normalized adjacency matrix and $W_{m},W_{s}$ are learnable weight matrices representing the message passing and skip-connection operations within the layer. The DiffPool model follows the same sequence of steps.

### 6.4 Evaluation Procedure

The best procedure for evaluating GNN pooling layers remains a matter of debate in the graph machine learning community. One may consider a fixed GNN architecture with a different pooling layer for each baseline; alternatively, the whole architecture can be optimized for each type of pooling layer. The first option, more akin to the typical procedure for evaluating pooling layers in CNNs on image domains, is used in papers like minCUT (Bianchi et al., 2019). The second option is more particular to GNNs and it is employed, for instance, by DiffPool (Ying et al., 2018). In this work, we choose the latter option for our evaluation.

We argue that for non-Euclidean domains, such as graph ones, the relationships between the nodes of the pooled graph and the ones of the input graph are semantically different from one pooling method to another. This is because pooling layers have different behaviors and may interact in various ways with the interleaved convolutional layers. Therefore, evaluating the same architecture with only the pooling layer(s) swapped is restrictive and might hide the benefits of certain operators. For example, Top-*k* pooling (one of our baselines) simply drops nodes from the input graph, instead of computing a smaller number of clusters from all nodes. Assume we fix the pooled graph to have only one node. Then Top-*k* would only select one node from the original graph. In contrast, DiffPool would combine the information from the entire graph in a single node. DiffPool would thus have access to additional information with respect to Top-*k*, so it would be unfair to conclude that one model is better than the other in such a setting. These differences implicitly affect the features of the output graph at that layer, which in turn affect the next pooling layer, as its computation depends on the features. This can have a cascading effect on the overall performance of the model. One might also argue that this procedure makes the evaluated models more homogeneous and, therefore, easier to compare. While this is true, the conclusions one can draw from such a comparison are much more limited because they are restricted to the particular architecture that was chosen.

For this reason, we have either run models with hyperparameters as previously reported by the authors, or optimized them ourselves end-to-end, where applicable. The best-performing configurations were (Appendix A details the hyperparameter search):

• MPR—learning rate $5e^{-4}$, hidden sizes {128,128} (except for {64,64} on IMDB-Binary and {32,32} on IMDB-Multi), interval overlap 25% on Proteins, Reddit-Binary, Mutag, IMDB-Multi and 10% otherwise, batch size 32 (except for 128 on Proteins) and:
• D&D, Collab, Reddit-Binary, Reddit-Multi-5K: cover sizes {20,5};
• Proteins, NCI1: cover sizes {8,2};
• Mutag, IMDB-Binary, IMDB-Multi: cover sizes {4,1};
• DMP—learning rate $5e^{-4}$, hidden sizes {128,128}, $\delta =1/{\left(\text{cluster}\_\text{size}\right)}^{2}$ and:
• Proteins: cover sizes {8,2}, batch size 128;
• Others: cover sizes {20,5}, batch size 32;
• Top-*k*—specific dataset configurations, as provided in the official GitHub repository^3^;
• minCUT—learning rate ${1\text{e}}^{-3}$, same architecture as reported by the authors in the original work (Bianchi et al., 2019);
• DiffPool—learning rate ${1\text{e}}^{-3}$, hidden size 32, two pooling steps, pooling ratio r=0.1 for D&D, Proteins, Collab and Reddit-Binary and r=0.25 for Mutag, NCI1, IMDB-Binary, IMDB-Multi and Reddit-Multi-5K, global average mean readout layer, with the exception of Collab and Reddit-Binary, where the hidden size was 128;
• Flat: hidden size 32.

### 6.5 Pooling Results

The graph classification performance obtained by these models is reported in Table 1. We reveal that MPR ranks either first or second on all social datasets, or achieves accuracy scores within 0.5% of the best-performing model. This result confirms that PageRank-based pooling exploits the power-law distributions in this domain. The performance of DMP is similar on social data and generally higher on molecular graphs. We attribute this to the fact that all nodes in molecular graphs tend to have a similar PageRank score—MPR is therefore likely to assign all nodes to one cluster, effectively performing a readout. In this domain, DMP performs particularly well on Mutag, where it is second-best and improves by 3.7% over MPR, showing the benefits of having a differentiable lens in challenging data settings. Overall, MPR achieves the best accuracy on two datasets (D&D, Collab) and the next best result on three more (Proteins, Reddit-Binary and Reddit-Multi-5k). DMP improves on MPR by less than 1% on NCI1, Proteins, IDMB-Binary and IMDB-Multi, showing the perhaps surprising strength of the simple, fixed-lens pooling MPR operator.

**TABLE 1:** Results obtained on classification benchmarks. Accuracy measures with 95% confidence intervals are reported. The highest result is bolded and the second highest is underlined. The first columns four are molecular graphs, while the others are social graphs. Our models perform competitively with other state of the art models.

Model	D&D	Mutag	NCI1	Proteins	Collab	IMDB-B	IMDB-M	Reddit-B	Reddit-5k
DMP (ours)	77.3±3.6	$\underline{84.0\pm 8.6}$	$\underline{70.4\pm 4.2}$	75.3±3.3	$\underline{81.4\pm 1.2}$	73.8±4.5	50.9±2.5	86.2±6.8	51.9±2.1
MPR (ours)	78.2±3.4	80.3±6.0	69.8±1.8	$\underline{75.2\pm 2.2}$	81.5±1.0	73.4±2.7	50.6±2.0	$\underline{86.3\pm 4.8}$	$\underline{52.3\pm 1.6}$
Top-*k*	75.1±2.2	82.5±6.8	67.9±2.3	74.8±3.0	75.0±1.1	69.6±3.8	45.0±2.8	79.4±7.4	48.5±1.1
minCUT	77.6±3.1	82.9±6.0	68.8±2.1	73.5±2.9	79.9±0.8	70.7±3.5	50.6±2.1	87.2±5.0	52.9±1.3
DiffPool	$\underline{77.9\pm 2.4}$	94.7±7.1	68.1±2.1	74.2±0.3	81.3±0.1	72.4±3.1	50.3±1.8	79.0±1.1	50.4±1.7
WL	77.4±2.6	74.5±6.5	76.4±2.7	74.7±3.2	78.5±1.1	72.1±3.1	$\underline{50.7\pm 2.9}$	66.7±10.4	49.2±1.4
Flat	69.9±2.2	71.8±4.3	65.5±1.7	70.2±2.6	80.9±1.4	$\underline{73.6\pm 4.2}$	48.5±2.4	70.0±10.8	49.5±1.7
Avg-MLP	63.7±1.4	69.1±5.8	55.7±2.8	61.8±1.7	74.8±1.3	71.5±2.9	49.5±2.2	53.6±6.2	45.9±1.6

## 7 Mapper for Visualisations

Graph pooling methods and summarized graph visualisations methods can be seen as two sides of the same coin, since both aim to condense the information in the graph. We now turn our attention to the latter.

### 7.1 Visualisations in Supervised Learning

**FIGURE 3:**
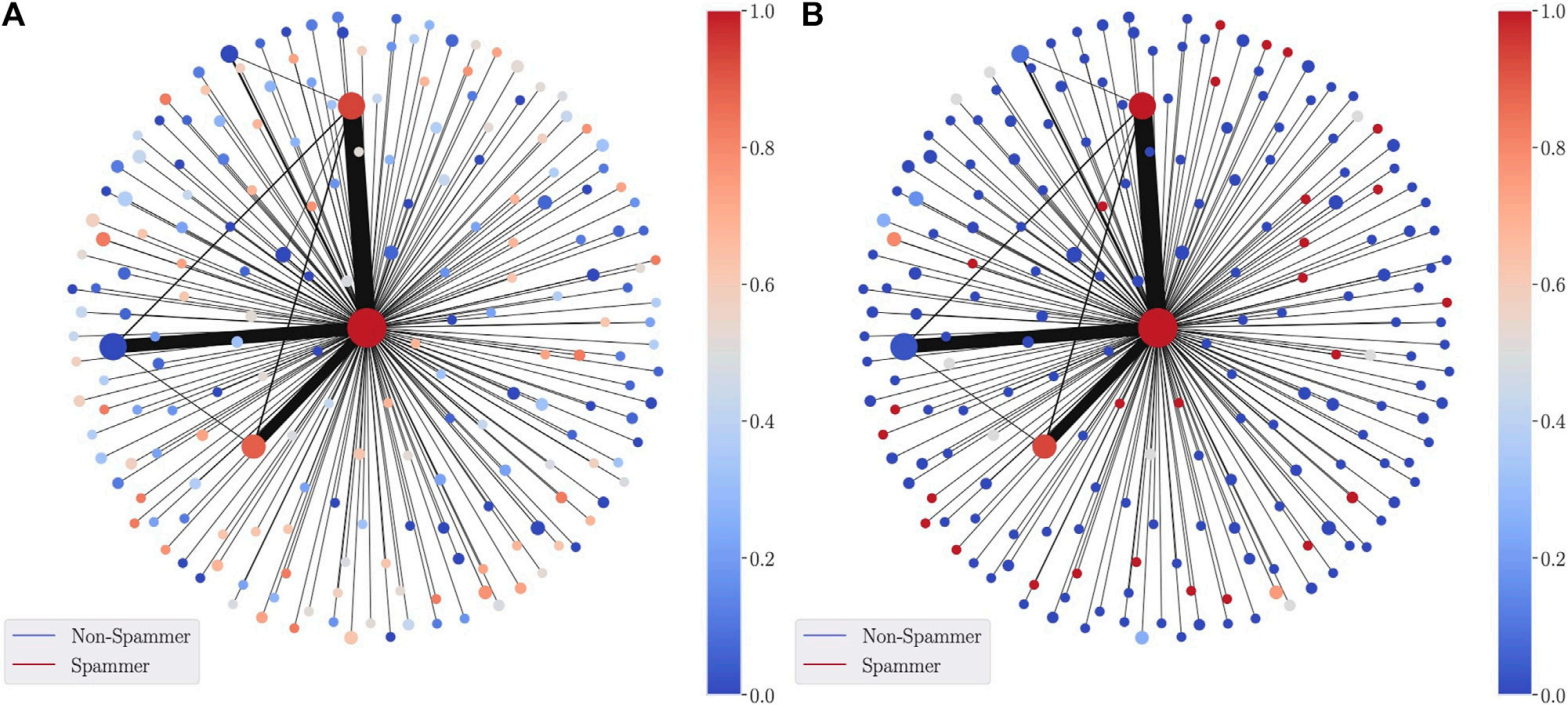
SDGM visualization using as a lens function the GNN-predicted probability of a node in the network to be Spam. The **(A)** is colored with the average predicted spam probability in each cluster, whereas the **(B)** is colored by the proportion of true spammers in each node.

The first application of DGM is in a supervised learning context, where $f_{\theta }$ is trained via a cross entropy loss function to classify the nodes of the graph. When the classification is binary, $f_{\theta }:V\to \left[0,1\right]$ outputs the probability that a node belongs to the positive class. This probability acts directly as the parameterization of the graph nodes. An example is shown in Figure 3 (left) for a synthetic dataset a network formed of spammers and non-spammers. Spammers are highly connected to many other nodes in the network, whereas non-spammers generally have fewer neighbors. For the lens function, we use a Graph Convolutional Network (GCN) (Kipf and Welling, 2016) with four layers (with 32,64,128,128 hidden units) and ReLU activations trained to classify the nodes of the graph. For the spammer graph, the lens is given by the predicted spam probability of each node and the cover consists of 10 intervals over [0,1], with 10% overlap.

Through the central cluster node, the SDGM visualization correctly shows how spammers occupy an essential place in the network, while non-spammers tend to form many smaller disconnected communities. When labels are available, we also produce visualisations augmented with ground-truth information. These visualisations can provide a label-driven understanding of the graph. For instance, in Figure 3 (right) we color each node of the SDGM visualization according to the most frequent class in the corresponding cluster. This second visualization, augmented with the ground-truth information, can also be used to compare with the model predictions.

### 7.2 Visualization in Unsupervised Learning

The second application corresponds to an unsupervised learning scenario, where the challenge is obtaining a parameterization of the graph in the absence of labels. This is the typical use case for unsupervised graph representation learning models (Chami et al., 2020). The approach we follow is to train a model to learn node embeddings in ${\mathbb{R}}^{d^{\prime }}$ (in our experiments, $d^{\prime }=512$), which can be reduced, as before, to a low-dimensional space via a dimensionality reduction method *r*. Unsupervised visualisations can be found in the qualitative evaluation in Section 7.3.

### 7.3 Qualitative Evaluation

In this section, we qualitatively compare SDGM against the two best-performing graph theoretic lens functions proposed by Hajij et al. (2018), on the Cora and CiteSeer (Sen et al., 2008) and PubMed (Yang et al., 2016) citation networks. Namely, we compare against a PageRank (Page et al., 1999) lens function and a graph density function $f\left(v\right)=\sum_{u\in V}\exp\left(\left(-D\left(u,v\right)/\delta \right)\right)$, where *D* is the distance matrix of the graph. For SDGM, we use a composition of an unsupervised Deep Graph Infomax (DGI) (Veličković et al., 2018) model $g_{\theta }:V\to {\mathbb{R}}^{512}$ and a dimensionality reduction function $r:{\mathbb{R}}^{512}\to {\mathbb{R}}^{2}$ based on *t*-SNE. To aid the comparison, we mark the nodes with the color of the most frequent class in the corresponding cluster. Additionally, we include a Graphviz (Gansner and North, 2000) plot of the full graph. We carefully fine-tuned the covers for each combination of model and graph.

**FIGURE 4:**
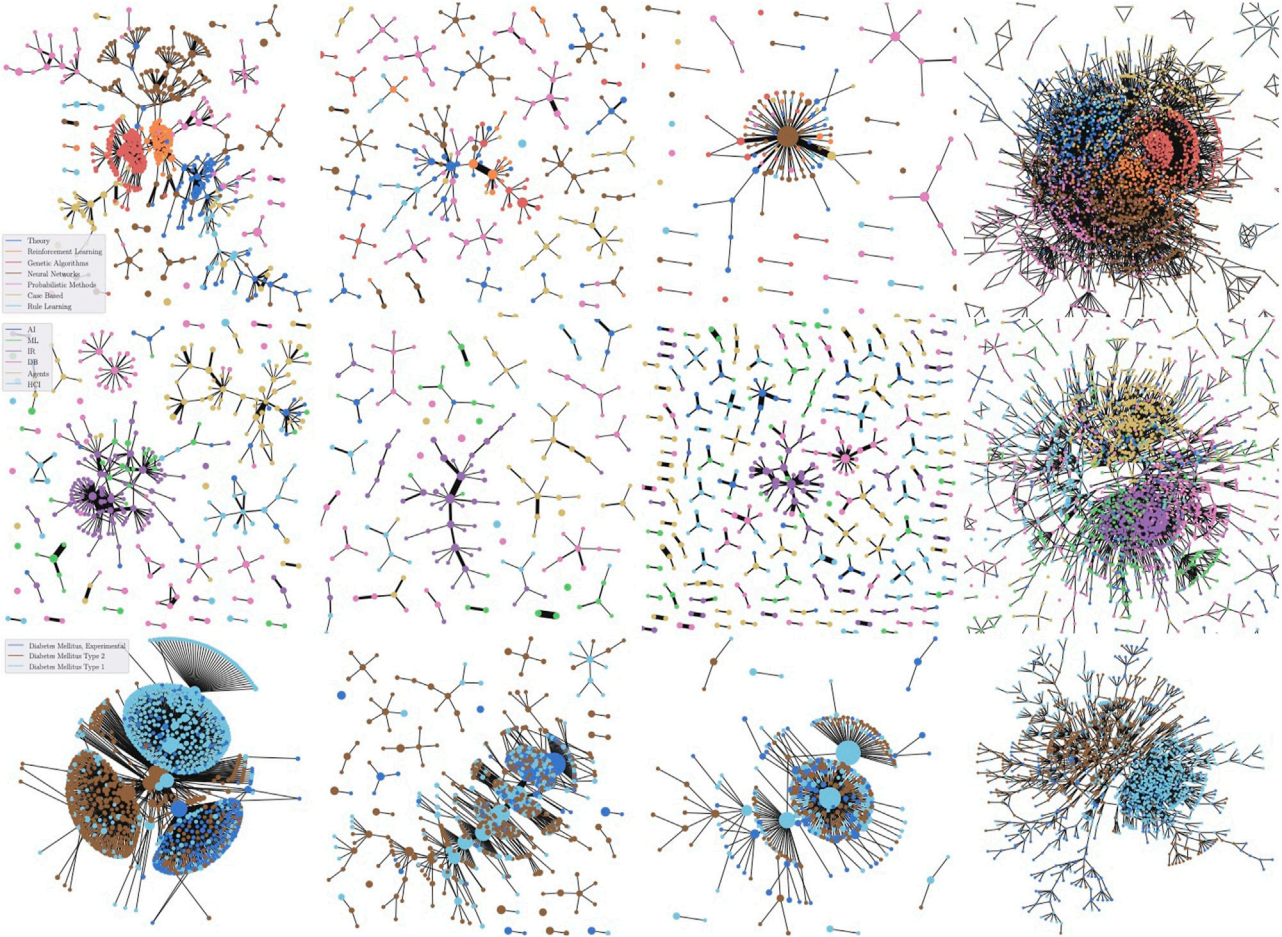
Qualitative comparison between SDGM (first column), Mapper with an RBF graph density function (Hajij et al., 2018) (second), and Mapper with a PageRank function (Hajij et al., 2018) (third). The Graphviz visualization of the graph cores (fourth column) are added for reference. The rows show plots for Cora, CiteSeer, and PubMed, respectively. The graphs are colored based on the most frequent class in each cluster to aid the comparison. SDGM with unsupervised lens implicitly makes all dataset classes appear in the visualization more clearly separated. This does not happen in the baseline visualisations, which mainly focus on the class with the highest number of nodes from each graph.

As depicted by Figure 4, SDGM successfully summarizes many of the properties of the graphs that are also reflected by full graph visualisations. For instance, on Cora, Genetic Algorithms (in dark orange) are shown to be primarily connected to Reinforcement Learning (orange). At the same time, related classes that largely overlap in the full visualisation—Probabilistic Methods and Neural Networks (NNs) on Cora or Information Retrieval (IR) and ML on CiteSeer—are connected in the SDGM plot. In contrast, the baselines do not have the same level of granularity and fail to capture many such properties. Both PageRank and the graph density function tend to focus on the classes with the highest number of nodes, such as the IR class on CiteSeer or the NNs class on Cora, while largely de-emphasizing other classes.

#### 7.3.1 Limitations

The proposed visualisations also present certain limitations. In an unsupervised learning setting, in the absence of any labels or attributes for coloring the graph, the nodes have to be colored based on a colourmap associated with the abstract embedding space, thus affecting the interpretability of the visualisations. In contrast, even though the graph theoretic lens functions produce lower quality visualisations, their semantics are clearly understood mathematically. This is, however, a drawback shared even by some of the most widely used data visualization methods, such as *t*-SNE or UMAP (McInnes et al., 2018). In what follows, we present additional visualisations and ablation studies.

### 7.4 Ablation Study for Dimensionality Reduction

**FIGURE 5:**
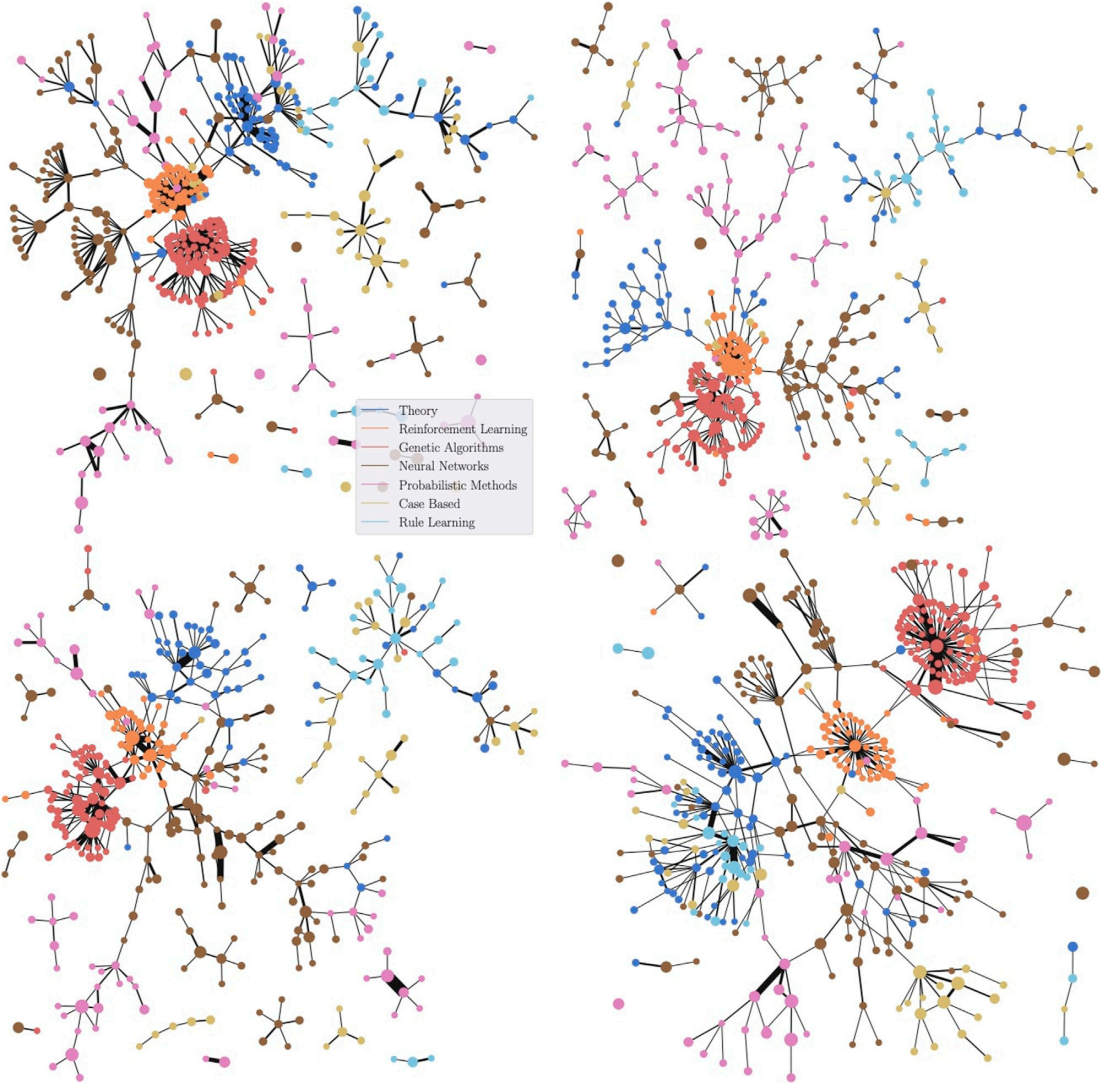
Ablation for dimensionality reduction methods; left–right, top–bottom: *t*-SNE, PCA, Isomap, UMAP. While *t*-SNE and UMAP produce slightly better visualisations, the graph features displayed by the visualisations are roughly consistent across all of the four dimensionality reduction techniques.

We study how the choice of the dimensionality reduction method for the unsupervised visualisations affects the output. To test this, we consider the following dimensionality reduction methods: *t*-SNE (van der Maaten and Hinton, 2008), UMAP (McInnes et al., 2018), IsoMap (Tenenbaum et al., 2000) and PCA. We use the same model as in Section 7.2 and Section 8. 2D cells for the cover of all models. The overlap was set after fine-tuning to 0.2 for *t*-SNE and UMAP, and to 0.1 for the other two models. Figure 5 displays the four visualisations. As expected, *t*-SNE and UMAP produce more visually pleasing outputs, due to their superior ability to capture variation in the GNN embedding space. However, the features highlighted by all visualisations are largely similar, generally indicating the same binary relations between clusters. This demonstrates that the GNN embedding space is robust to the choice of the dimensionality reduction method.

### 7.5 Ablation for the Unsupervised Lens

To better understand the impact of GNNs on improving the quality of the Mapper visualisations, we perform an ablation study on the type of unsupervised lens functions used within Mapper. The first model we consider is simply the identity function taking as input only graph features. The second model is a randomly initialized DGI model. Despite the apparent simplicity of a randomly initialized model, it was shown that such a method produces reasonably good embeddings, often outperforming other more sophisticated baselines (Veličković et al., 2018). Finally, we use our trained DGI model from Section 7.2. For all models, we perform a *t*-SNE reduction of their embedding space to obtain a 2D output space and use 81 overlapping cells that cover this space. An overlap of 0.2 is used across all models.

**FIGURE 6:**
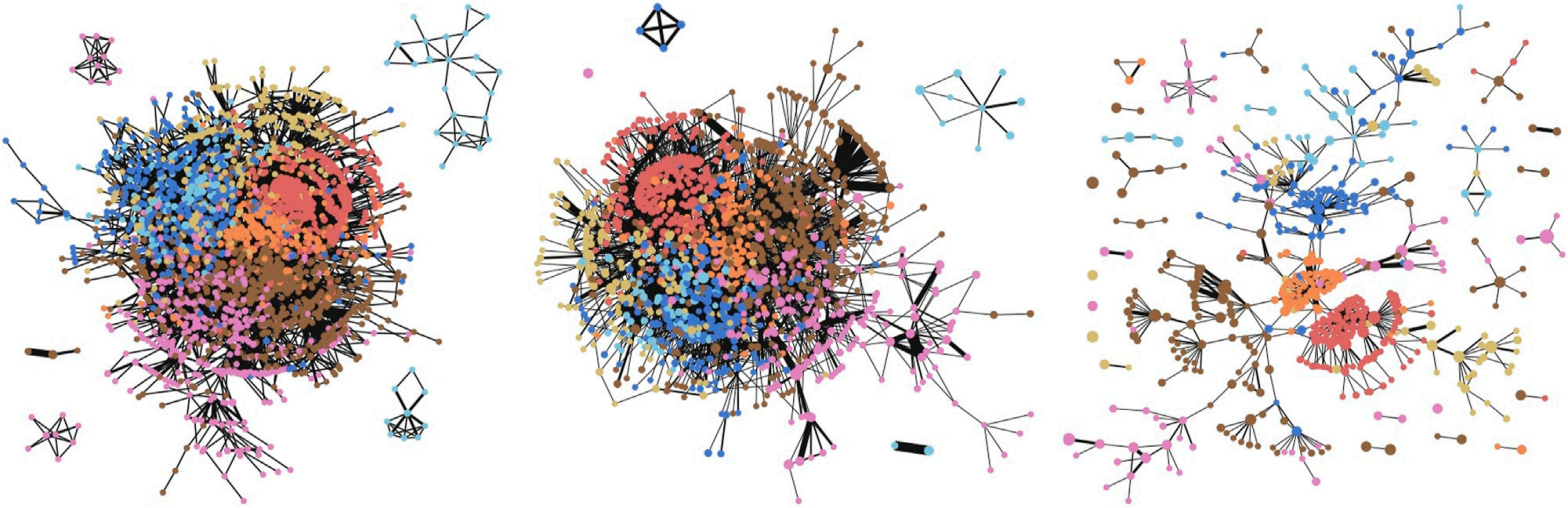
Ablation for different types of unsupervised lenses (identity, untrained DGI, trained DGI). The trained DGI model significantly improves the quality of the visualisations.

The three resulting visualisations are depicted in Figure 6. The identity model and the untrained DGI model do not manage to exploit the dataset structure and neither does particularly well. In contrast, the trained DGI model emphasizes all the classes in the visualization, together with their main interactions.

### 7.6 Hierarchical Visualisations

**FIGURE 7:**
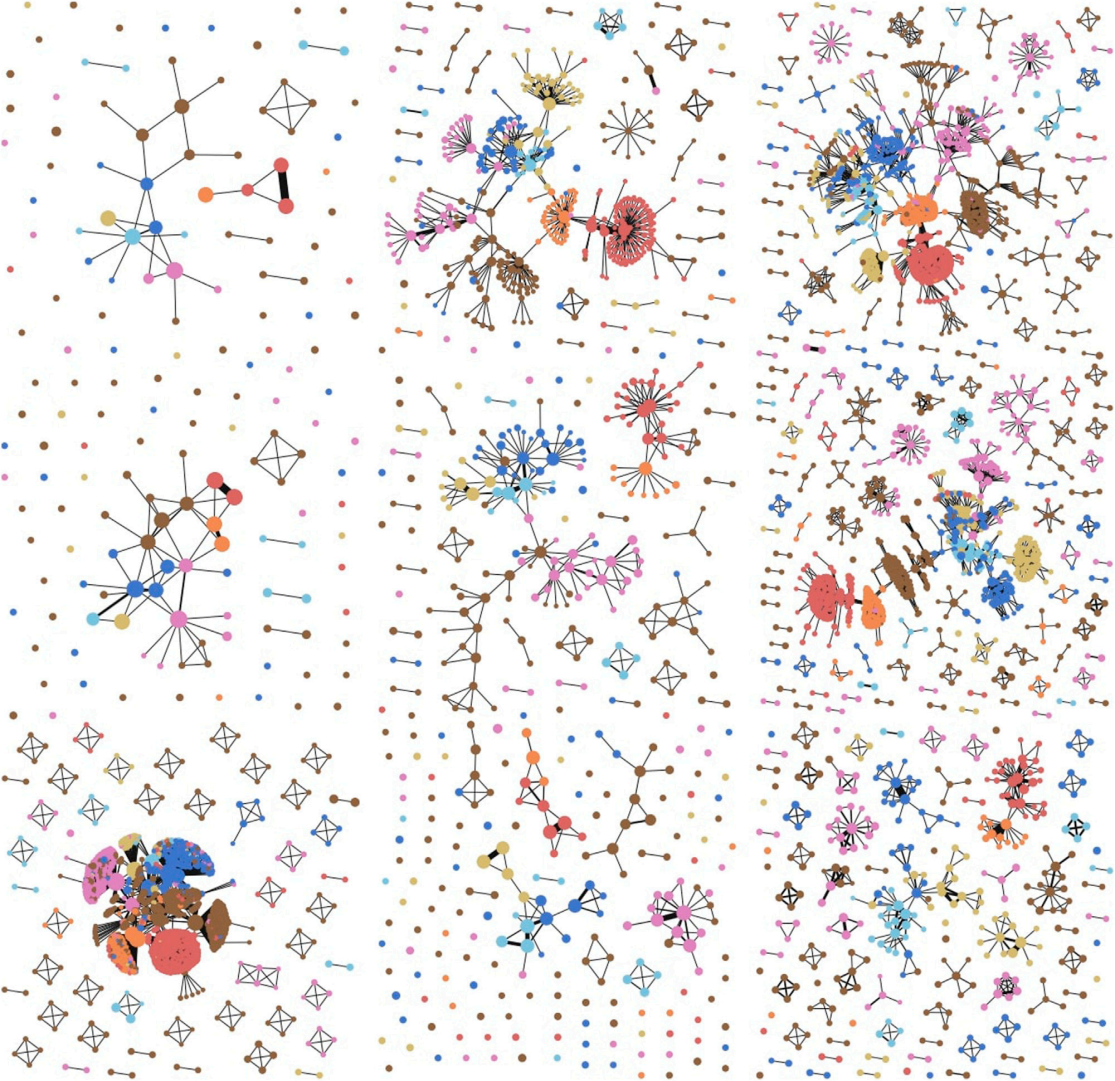
Hierarchical visualisations of the Cora citation network using various number of cover cells and degrees of overlap. Rows **(top–bottom)** have a different overlap (*g*) between intervals: g=0.1, g=0.25, g=0.35; columns (left–right): n=16, n=64, n=256.

One of the most powerful features of Mapper is the ability to produce multi-resolution visualisations through the flexibility offered by the cover hyperparameters. As described in Section 4, having a higher number of cells covering the output space results in more granular visualisations containing more nodes, while a higher overlap between these cells results in increased connectivity. We highlight these trade-offs in Figure 7, where we visualize the Cora citation network using nine combinations of cells and overlaps. These kinds of hierarchical visualisations can help one identify the persistent features of the graph. For instance, when inspecting the plots that use n=64 cells, the connections between the light blue class and the yellow class persist for all 3 degrees of overlap, which indicates that this is a persistent feature of the graph. In contrast, the connection between the red and orange classes is relatively reduced (g=0.25) or none (g=0.1) for low values of overlap, but it clearly appears at g=0.35 in the top-right corner, suggesting that the semantic similarity between the two classes is very scale-sensitive (that is, less persistent).

### 7.7 The Importance of Capturing Structural Information

**FIGURE 8:**
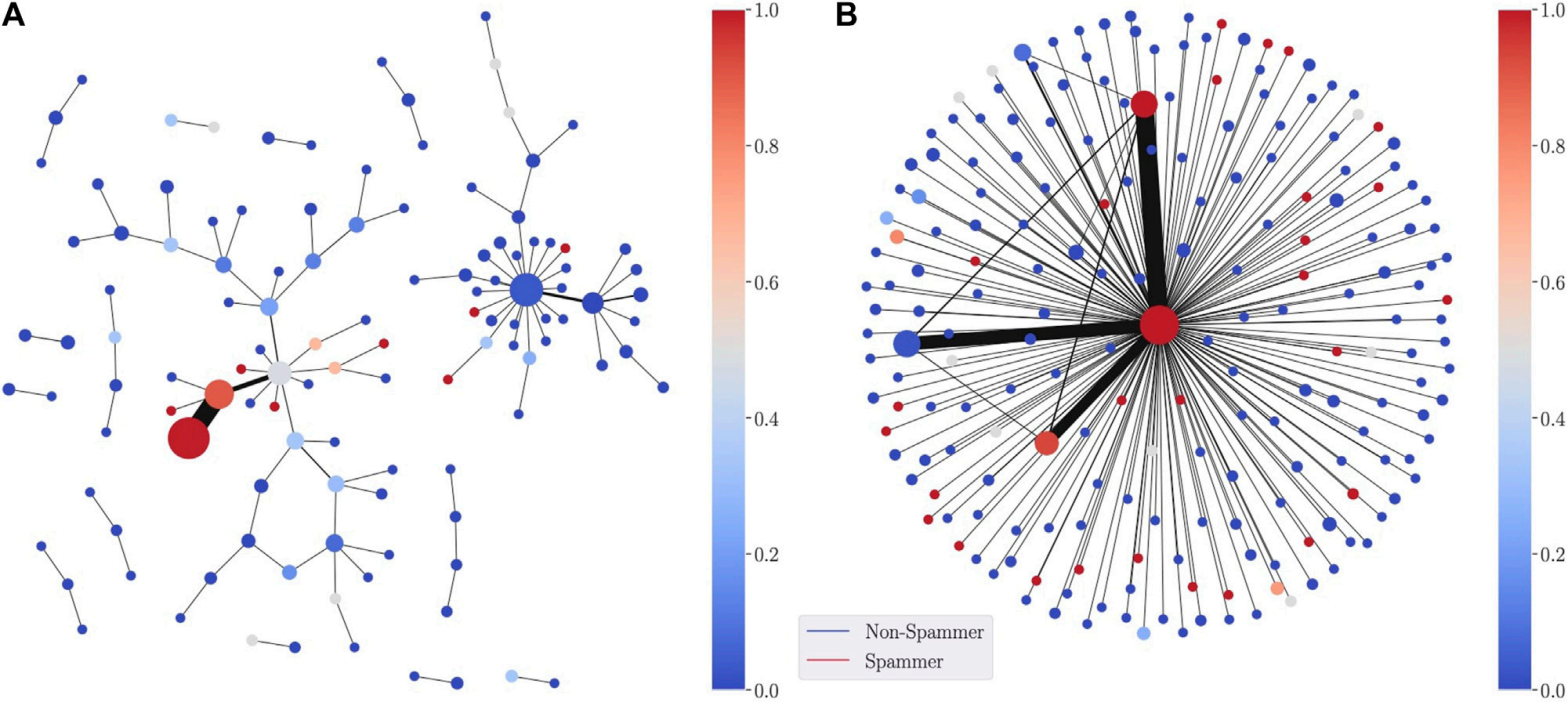
DGM **(A)** vs SDGM **(B)** visualization of the sythetic spammer datasets. DGM does not capture important relational information between spammers and non-spammers.

In this section, we revisit the synthetic spammer dataset to illustrate the importance of capturing structural information via the edge-refined pull back cover operator. To that end, we compare SDGM with a version using the usual refined pull back cover as in Hajij et al. (2018), while using the same lens function for both (a GCN classifier). We refer to the latter as DGM. The visualisations produced by the two models are included in Figure 8. We note that while both models capture the large cluster of spammers at the center of the network and the smaller communities of non-spammers, DGM does not capture the structural relationships between spammers and non spammers since it encodes only semantic relations.

## 8 Conclusion

We have introduced Deep Graph Mapper, a topologically grounded method for producing informative graph visualisations with the help of GNNs. We have shown these visualisations are not only useful for understanding various graph properties, but can also aid in visually identifying classification mistakes. Additionally, we have proved that Mapper is a generalization of soft cluster assignment methods, effectively providing a bridge between graph pooling and the TDA literature. Based on this connection, we have proposed two Mapper-based pooling operators: a simple one that scores nodes using PageRank and a differentiable one that uses RBF kernels to simulate the cover. Our experiments show that both layers yield architectures competitive with several state-of-the-art methods on graph classification benchmarks.

## Data Availability

The original contributions presented in the study are included in the article/Supplementary Material, further inquiries can be directed to the corresponding authors.

## Footnotes

1 Code to reproduce models and experimental results is available at https://github.com/crisbodnar/dgm.

2 Note that one or more {embedding → pooling} operations may be sequentially performed in the pipeline.

3
https://github.com/HongyangGao/Graph-U-Nets/blob/48aa171b16964a2466fceaf4cb06fc940d649294/run_GUNet.sh

## Appendix

### A Model Architecture and Hyperparameters.

We additionally performed a hyperparameter search for DiffPool on hidden sizes 32,64,128 and for DGM, over the following sets of possible values:

• all datasets: cover sizes {[40,10],[20,5]}, interval overlap {10%,25%};
• D&D: learning rate $\left\{5e^{-4},1e^{-3}\right\}$;
• Proteins: learning rate $\left\{2e^{-4},5e^{-4},1e^{-3}\right\}$, cover sizes {[24,6],[16,4],[12,3],[8,2]}, hidden sizes {64,128}.
